# Loss of miR-10a Activates *Lpo* and Collaborates with Activated Wnt Signaling in Inducing Intestinal Neoplasia in Female Mice

**DOI:** 10.1371/journal.pgen.1003913

**Published:** 2013-10-24

**Authors:** Gustavo Stadthagen, Disa Tehler, Nina Molin Høyland-Kroghsbo, Jiayu Wen, Anders Krogh, Klaus T. Jensen, Eric Santoni-Rugiu, Lars H. Engelholm, Anders H. Lund

**Affiliations:** 1 Biotech Research and Innovation Centre (BRIC), University of Copenhagen, Copenhagen, Denmark; 2 Bioinformatics Centre Department of Biology, University of Copenhagen, Copenhagen, Denmark; 3 Department of Pathology, Diagnostic Centre, Rigshospitalet, Copenhagen, Denmark; 4 The Finsen Laboratory, Rigshospitalet, Faculty of Health Sciences, University of Copenhagen, Copenhagen, Denmark; University of Washington, United States of America

## Abstract

miRNAs are small regulatory RNAs that, due to their considerable potential to target a wide range of mRNAs, are implicated in essentially all biological process, including cancer. miR-10a is particularly interesting considering its conserved location in the *Hox* cluster of developmental regulators. A role for this microRNA has been described in developmental regulation as well as for various cancers. However, previous miR-10a studies are exclusively based on transient knockdowns of this miRNA and to extensively study miR-10a loss we have generated a *miR-10a* knock out mouse. Here we show that, in the *Apc^min^* mouse model of intestinal neoplasia, female miR-10a deficient mice develop significantly more adenomas than miR-10^+/+^ and male controls. We further found that *Lpo* is extensively upregulated in the intestinal epithelium of mice deprived of miR-10a. Using *in vitro* assays, we demonstrate that the primary miR-10a target KLF4 can upregulate transcription of *Lpo*, whereas siRNA knockdown of KLF4 reduces LPO levels in HCT-116 cells. Furthermore, Klf4 is upregulated in the intestines of *miR-10a* knockout mice. Lpo has previously been shown to have the capacity to oxidize estrogens into potent depurinating mutagens, creating an instable genomic environment that can cause initiation of cancer. Therefore, we postulate that *Lpo* upregulation in the intestinal epithelium of miR-10a deficient mice together with the predominant abundance of estrogens in female animals mainly accounts for the sex-related cancer phenotype we observed. This suggests that miR-10a could be used as a potent diagnostic marker for discovering groups of women that are at high risk of developing colorectal carcinoma, which today is one of the leading causes of cancer-related deaths.

## Author Summary

Posttranscriptional regulation by microRNA molecules constitutes an important mechanism for gene regulation and numerous studies have demonstrated a correlation between deregulated microRNA levels and diseases, such as cancer. However, genetics studies linking individual microRNAs to the etiology of cancer remain scarce. Here, we provide causal evidence for the involvement of the conserved microRNA miR-10a in the development of intestinal adenomas in the face of activated Wnt signaling. Interestingly, we find that loss of miR-10a mediates an increase in intestinal adenomas in female mice only and delineate the pathway to involve aberrant upregulation of the miR-10a target Klf4 and subsequent transcriptional activation of the *Lpo* gene encoding the antibacterial protein Lactoperoxidase. Lpo, in turn, has previously been demonstrated to oxidize estrogens into DNA-damaging mutagens.

## Introduction

A growing number of studies show the importance of aberrant miRNA expression in cancer. Although miRNA profiling studies have proven useful in defining signatures of cancer-deregulated miRNAs with diagnostic and/or prognostic value [1], [2], establishing casual relationships is not always possible. Altered miRNA expression in cancer can arise from genomic abnormalities but also by alteration of upstream regulators of miRNA expression and/or maturation, including epigenetic silencing [3].

The *miR-10* miRNA family members are encoded in evolutionarily conserved loci within the Homeobox (*Hox*) gene clusters of developmental regulators [4], [5]. Co-expression of *miR-10* and *Hox* genes during development [6], [7] and experimental evidence of miR-10 targeting of *HOX* transcripts [8]–[10] has suggested a role for this miRNA family in development. Mammalian *miR-10a* and *miR-10b* are located upstream from *HoxB4* and *HoxD4* respectively and they present a very high degree of sequence conservation, differing at their eleventh nucleotide only (U and A respectively), which thermodynamically enables them to target a fully overlapping set of mRNAs [11], . Importantly, both up- and downregulation of miR-10 has been reported in several cancers and although the number of studies where such deregulation was causally linked to the pathogenesis of cancer remains scarce (for a review, see [4]), some miR-10 targets have been demonstrated to be mechanistically linked to metastasis, invasion and migration as well as cell proliferation [9], [10], [13]–[16].

Colorectal cancer is the second most commonly diagnosed cancer in women and it is one of the leading causes of cancer-related deaths in the world [17]. Colon cancer arises from the epithelial cells of the lumen of the colon where benign adenomatous polyps are established as an initial step. These further progresses into more advanced adenomas showing high-grade dysplasia and can ultimately evolve into invasive cancer. One of the most studied causes of colon cancer is aberrant signaling of the evolutionary conserved Wnt pathway, which is tightly regulated during development and crucial for adult tissue homeostasis in the intestinal tract [18]. The tumor suppressor Adenomatous polyposis coli (*Apc*) gene is an essential negative regulator of the Wnt pathway and loss of function of this gene is associated with a great majority of colorectal cancers [19]–[21]. Clinically, colon cancer is categorized in four stages (I to IV) corresponding to its degree of progression [22], [23]. Chromosomal instability, DNA-repair and aberrant DNA methylation have been shown to be important for the development and progression of colon cancer (for a review, see [22]). Interestingly, miR-10a was previously found to be moderately up regulated in solid tumors and stage II but not in stage I cancers in the colon [24], [25].

Here we have generated a *miR-10a* knock out (KO) mouse and crossed it with the *Apc^Min^* colon cancer mouse model of familial adenomatous polyposis. Interestingly, only in female mice the specific lack of miR-10a sensitized the intestinal epithelium to increased tumor development. Lactoperoxidase (*Lpo*) was strikingly deregulated between *miR-10a* KO and WT intestines and our data suggests that *LPO* is an indirect target of miR-10a, being directly regulated by the transcription factor and primary miR-10a target KLF4. Compellingly, Lpo has previously been reported to have the capacity to oxidize estrogenic substrates into potent depurinating mutagens, which are known to contribute to the initiation of cancer.

## Results

### Generation of *miR-10a* KO mice

**Figure 1:**
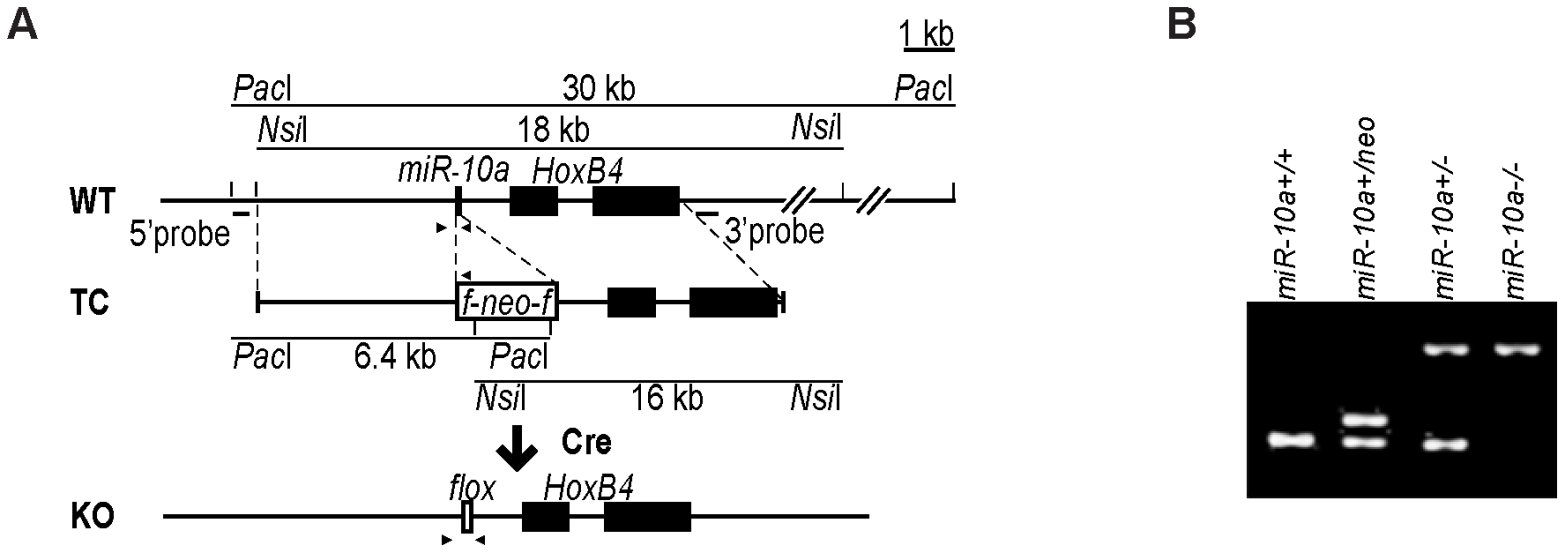
Generation of *miR-10a* KO mice. (A) Schematic representation of the *miR-10a* WT locus, the targeting construct used for inactivation and the final *miR-10a null* allele. The targeting construct (TC) harbored a *miR-10a* inactivated allele, where 70 nucleotides from the pre-miRNA sequence were replaced with a neomycin resistance cassette (*neo*) flanked by *loxP* sites and long homologous regions for recombination. To obtain the final *miR-10a null* allele (KO), the neomycin cassette was removed in the mouse germ line by breeding heterozygous mice to transgenic mice harboring the *Cre* transgene. Arrowheads depict the sites recognized by different primers used in genotyping of mice. (B) Genotyping PCR of mice with all different *miR-10a* genotypes generated. Primers L_chkinsrtmiR10a.5d and 10a.internal amplified a 273 bp fragment corresponding to the *miR-10* WT allele and 361 bp for the floxed *miR-10a* KO allele, L_chkinsrtmiR10a.5d and R_chkinsrtmiR10a.5 amplified 291 bp from the *miR-10a^neo^* allele. The location of all these primers is depicted in (A).

To assess the physiological role and pathophysiological significance of miR-10a, we generated a null allele of *miR-10a* by gene targeting. The targeted locus consisted of a *loxP*-flanked *neo* selection cassette, which replaced the 70 central nucleotides of the pre-miRNA sequence of *miR-10a* (Figure 1A). The targeting vector was introduced into embryonic stem (ES) cells, selected with G418 and correctly targeted clones with the genotype *miR-10a^+/neo^* were identified by Southern blotting (data not shown). Chimeric mice were generated that transmitted the mutated allele through the germ line. All offspring were genotyped and verified by PCR (Figure 1B). Breeding of *miR-10a^+/neo^* mice to a mouse strain holding an ubiquitously expressed *Cre* recombinase transgene [26] resulted in deletion of the *miR-10a* genomic sequence and its replacement by a residual *LoxP* site, yielding mice with the *miR-10a^+/−^* genotype (Figure 1B). Mice carrying the *miR-10a* floxed allele (*miR-10a* ^−^) were intercrossed with C57BL/6 mice for at least 7 generations before generating experimental cohorts.

Interbreeding of heterozygous *miR-10a^+/−^* mice produced homozygous null (*miR-10a^−/−^*) offspring at the expected Mendelian ratios (Figure S1A). These mice were indistinguishable from littermate controls in terms of growth and development (Figure S1B) and did not show decreased survival or an increased incidence of spontaneous tumor development compared to WT mice by 2 years of age. Likewise, gross pathological examination of the major organs revealed no differences and analyses of embryo fibroblast cultures did not show differences in proliferation rates or time to replicative senescence (data not shown). To confirm that the mutant allele was null, quantitative RT-PCR was performed on RNAs extracted from the intestines of WT and homozygous (*miR-10a^−/−^*) mutant mice (Figure S1C). qRT-PCR using specific primers for miR-10b on the same RNA showed no significant difference in the level of this close member of the miR-10 family, suggesting no occurrence of dose-dependent compensation via trans-regulation of miR-10b in the absence of miR-10a (Figure S1D). *HoxB4* is located 992 nucleotides downstream from the *miR-10a* gene and the transcription of both genes has been proposed to be co-regulated [6], [7]. Deletion of *miR-10a* did not interfere with transcription of *HoxB4* since similar levels of *HoxB4* mRNA were detected by quantitative RT-PCR in intestinal samples (Figure S1E).

### *miR-10a* deficiency enhances intestinal tumorigensis in female *Apc^Min^* mice

Since *miR-10a* inactivation alone did not give rise to increased spontaneous tumor formation, we evaluated if the lack of this miRNA could modify tumor formation upon an additional oncogenic injury. Profiling of WT mouse tissues for miR-10a and miR-10b revealed that miR-10a was relatively highly expressed in the mouse intestinal tract (Figure S2). Furthermore, profiling studies have shown that miR-10a expression is deregulated in human colon cancer [9], [24], [25]. Therefore, the *Apc^Min^* mouse model [27] was chosen to evaluate the role of miR-10a in intestinal neoplasia development. *Apc^Min^* mice carry a mutation in the murine homolog of the human *APC* gene [28] and develop multiple intestinal tubular adenomas similar to those found in patients with the familial adenomatous polyposis syndrome. Furthermore, the *Apc^Min^* mouse model is frequently used to evaluate the significance of genetic modifiers [29]–[32].

**Figure 2:**
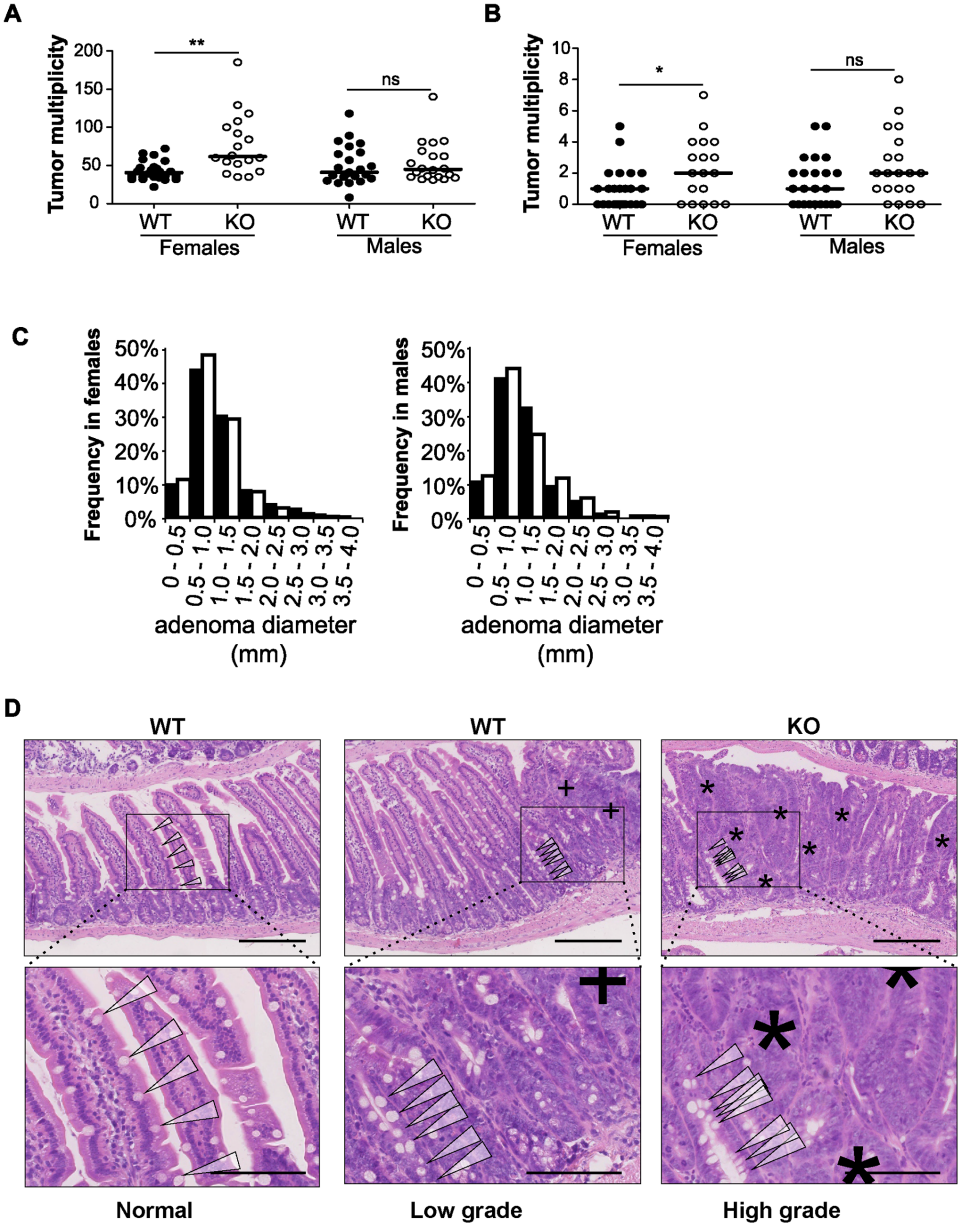
Disruption of *miR-10a* leads to enhanced intestinal tumorigenesis in *Apc^Min^* mice. Tumor multiplicity in the small (A) and large intestines (B) of female and male *miR-10a* ^+/+^;*Apc^Min^* (WT; n = 22 and n = 19 for each sex) and *miR-10a^−/−^*;*Apc^Min^* (KO; n = 15 and n = 16 for each sex) mice; each dot represents data for one mouse. Mean adenoma multiplicities per mouse for each group were: WT = 41.95 and KO = 79.33 for female mice and WT = 50.37 and KO = 55.19 for male mice in the small intestine and WT = 0.82 and KO = 2.40 for female mice and WT = 1.47 and KO = 2.44 for male mice in the large intestine. * p = 0.014, ** p = 0.0042 (two-tailed *t*-test) (C) Size distribution of polyps in the small intestine of female and male *miR-10a* ^+/+^;*Apc^Min^* (WT; filled bars) and *miR-10a^−/−^*;*Apc^Min^* (KO; empty bars) mice. Mean tumor diameters were 1.01 and 1.04 mm for males WT and KO respectively and 1.03 and 0.94 for females WT and KO respectively (*p* = 0.782 and *p* = 0.4113, Wilcoxon rank sum test). (D) Left panel shows normal appearing small intestine with characteristic villi and well-ordered distribution of goblet cells together with basal location of epithelial nuclei. Middle panel is a typical example of a low-grade dysplasia in *miR-10a* ^+/+^;*Apc^Min^* (WT) mice with accumulation of irregular goblet cells pattern (arrowheads) and some loss of nuclear polarity (indicated by “+”). Right panel shows a typical *miR-10a^−/−^*;*Apc^Min^* (KO) high-grade dysplasia with a large area of loss of goblet cells, widespread loss of nuclear polarity, nuclear pleomorphism, and almost complete loss of villus organization (indicated by “*”). Note the transition from lower-grade dysplasia area with highly irregular goblet cell distribution (arrowheads). Scalebar = 100 µM. Whole intestines were paraffin-embedded as “Swiss rolls”, sectioned and stained with hematoxylin and eosin. All animals were in a B6 background and between 110 and 160 days of age.

To examine the impact of disrupting *miR-10a* in *Apc^Min^* mice, the *miR-10a* KO and *Apc^Min^* mouse strains (both in a C57BL/6 background) were intercrossed. Mice were sacrificed at around 140 days of age for tumor burden evaluation. Strikingly, the mean tumor multiplicity in small intestines of *miR-10a^−/−^;Apc^Min^* female mice (79.33, n = 15) was almost twice as high as in corresponding *miR-10a^+/+^;Apc^Min^* age matched controls (41.95, n = 22) (p = 0.0042) (Figure 2A). Tumor multiplicity (TM) and tumor incidence (TI) in the large intestine were also higher in the female mice lacking miR-10a (TM = 2.40, TI = 72.2%), compared to control (TM = 0.82, TI = 54.2%) but those differences were less significant or not significant, respectively (p*_TM_* = 0.014; p_TI_ = 0.38; Figure 2B). Noteworthy, the *miR-10a* genotype did not affect tumor multiplicity in *Apc^Min^* male mice irrespective of the anatomic location (p_small intestine_ = 0.61, p_large intestine_ = 0.16; Figures 2A and 2B). The incidence of polyps in the large intestine of male mice also remained unaffected (p = 0.30).

To examine the effect of miR-10a deficiency on tumor size, the flat adenomas of the small intestine were measured at their largest diameter, and this measure was used as indicator of tumor size. No significant difference was observed in mean tumor diameters between *miR-10a^−/−^*;*Apc^Min^* and *miR-10a^+/+^*;*Apc^Min^* control mice irrespective of gender (p = 0.614 for males and p = 0.071 for females; Figure 2C).

Entire intestinal tracts were paraffin-embedded as “Swiss rolls” and hematoxylin and eosin (H&E) stained sections were examined microscopically based on pathological criteria. Qualitatively, compared to *miR-10a^+/+^*;*Apc^Min^* mice intestines, samples from *miR-10a^−/−^*;*Apc^Min^* mice presented more frequently adenomas with high-grade dysplasia and a higher incidence of tubulo-villous adenomas, these differences were more evident in female mice (Figure 2D). However, no invasive carcinomatous processes were observed in any of the analyzed samples. The increased tumor multiplicity and colonic epithelial dysplasia along with unaffected adenoma sizes in the absence of *miR-10a*, suggest that miR-10a is involved in the tumor initiation/promotion steps but not in enhancing cell proliferation in the *Apc^Min^* model of intestinal neoplasia [33], [34]. However, due to ethical constraints, the mice are sacrificed at a relatively young age and we cannot formally rule out that an effect on tumor progression would be discernable in the end-stage tumors.

### Identification of miR-10a targets in the intestines of female mice

miRNA exert their biological functions primarily by regulating the translation and stability of targeted mRNAs [35], [36]. Microarray analysis of deregulated transcripts upon alteration of individual miRNAs in cells and tissues has been proven as a useful tool for identifying direct and indirect miRNA targets [37], [38]. Therefore, colon mRNA expression was analyzed in *miR-10a* KO and WT female mice using Affymetrix microarrays. Although 452 transcripts were significantly deregulated in the *miR-10a* KO samples compared to WT (P≤0.05), the levels of up- or downregulation were modest and only three protein coding genes had false discovery rates (FDR) lower than 15% (Table S2). In addition, we did not detect any enrichment for predicted miR-10a targets among the deregulated transcripts. Although adaptation to loss of miR-10a or functional redundancy by the remaining miR-10b could account for the invariable levels of miR-10 intestinal targets in the absence of *miR-10a*, low levels of mRNA deregulation upon miRNA alterations have been previously observed [39], [40]. Furthermore, the variation inherent to tissue samples may shadow a high deregulation within a specific cell type of the tissue. With the exception of *Lpo*, qRT-PCR measurement of selected transcripts, using independent sample sets, did not show any consistent deregulation of genes identified by the microarray as variant between genotypes. Transcript abundance of selected oncogenes and tumor suppressors, relevant in intestinal tumorigenesis or previously predicted as miR-10a targets but not detected in the microarray, were also unchanged in *miR-10a* deficient compared to WT intestines (Figure S3).

### *Lpo* is a secondary target of miR-10a in the intestines of female mice and in human cell lines

**Figure 3:**
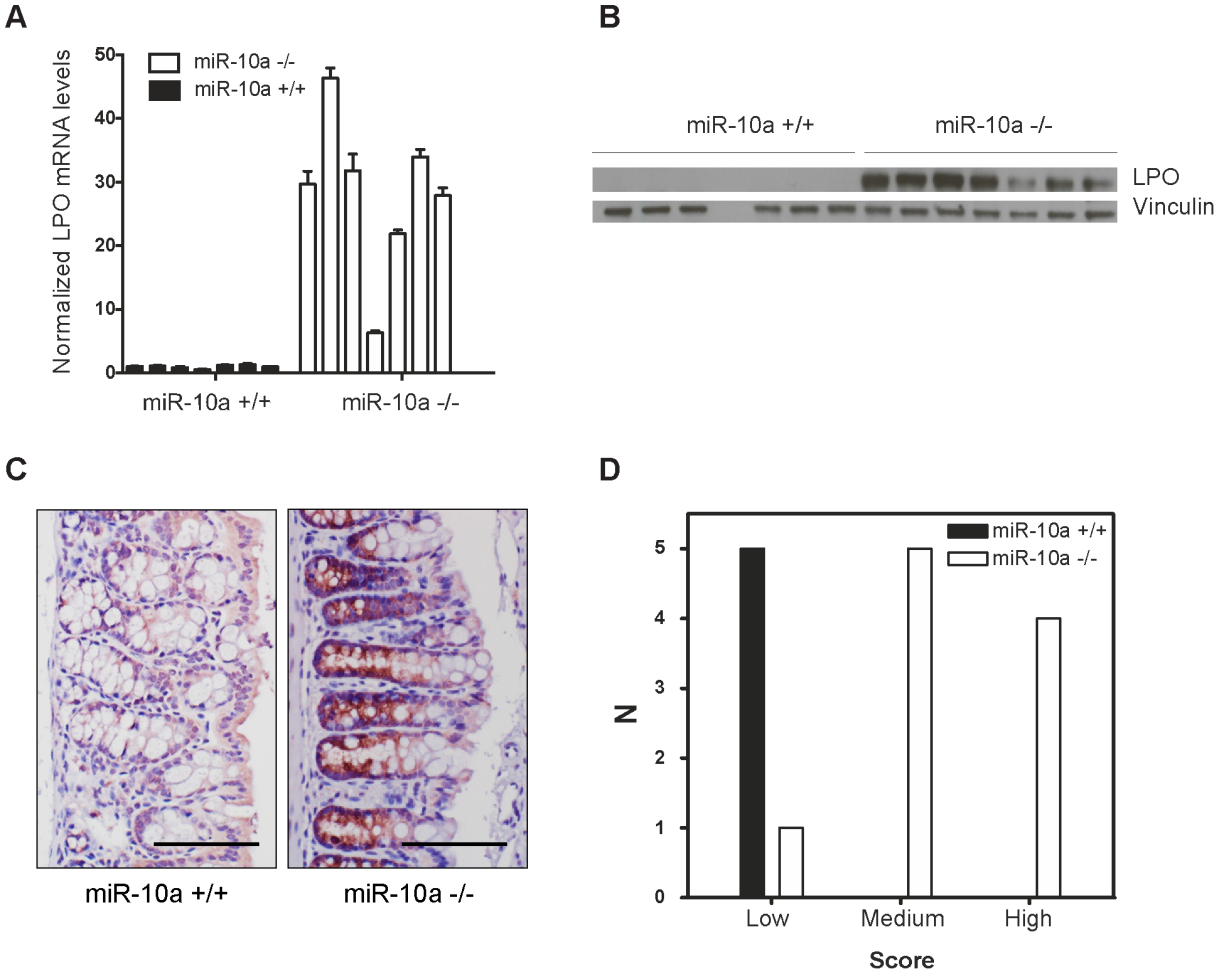
*Lpo* is transcriptionally upregulated in the intestines of *miR-10a* deficient female mice. (A) *Lpo* mRNA is ∼29-fold upregulated in intestines of *miR-10a* KO compared to WT mice as shown by qRT-PCR. *Lpo* mRNA levels are normalized to *Actb* and values ± SD are shown relative to the first WT sample. (B) Western-blot from same tissue samples as in (A) confirming upregulation on protein level. Vinculin was used as loading control. As evident from Lpo and Vinculin control as well as ponceau staining (now shown), sample 4 did not contain any protein for unknown reason. (C) Representative immunohistochemistry staining of Lpo in *miR-10a* WT and KO intestine. Scale bar 100 µm. (D) Scoring of Lpo expression level estimated by distribution and staining intensity in Lpo stained intestines of WT and miR-10a^−/−^ mice. Scoring is divided into low, medium or high expression. Consistent with qRT-PCR and Western blotting analysis a significant difference (*p*≤0.006, Pearson chi-square test with exact probability) in Lpo expression is observed between the different genotypes.

Interestingly, *Lpo* was identified as exceptionally highly upregulated in the intestines of *miR-10a* KO female mice, displaying a 9.44 fold increase in expression compared to WT (p = 1.1e-6; adjusted for multiple testing). Lactoperoxidase normally plays a role in antimicrobial defense and removal of toxic hydrogen peroxide [41], [42]. However, this enzyme has also been shown to catalyze the activation of endogenous and xenobiotic compounds, such as estrogens and arylamines, into potent depurinating mutagens [43]–[46]. By increasing genome instability, LPO has been proposed to exert a pro-oncogenic role in tissues like the mammary gland [43], [47]. Given the importance of genome stability in the initiation and progression of intestinal tumorigenesis [48], a similar mechanism might be involved in the phenotype observed in *miR-10a^−/−^*;*Apc^Min^* female mice, i.e. the upregulation of Lpo in *miR-10a* KO mice would enhance estrogen oncogenic activation, leading to a highly instable genomic environment. qRT-PCR confirmed *Lpo* overexpression by 29-fold in female *miR-10a* KO intestines compared to WT (Figure 3A). Similar degrees of *Lpo* upregulation were obtained in male *miR-10a* KO mice (data not shown). Accordingly, analysis of protein extracts from *miR-10a* KO and WT intestines equally revealed a strong induction of Lpo in *miR-10a* deficient samples (Figure 3B). In agreement with the analysis of *Lpo* mRNA and Lpo protein level, a clear difference in both intensity and distribution area of Lpo staining was observed between *miR-10a* KO and WT mice (p≤0.006, Pearson chi-square test with exact probability). Consistently, all stained WT samples had faint Lpo signal in a limited area of the intestine thus scoring low expression while the majority of *miR-10a* KO tissue samples had a significantly more intense Lpo signal and a more widespread Lpo expression pattern, thus scoring medium to high (Figure 3C and 3D).

No *bona fide* miR-10a binding sites could be identified in the 3′ UTR of *Lpo* but cryptic sites in the 5′ UTR and coding sequence (CDS) carried significant complementarity to miR-10a (Figure S4A). To determine whether *Lpo* was a direct target of miR-10a, via the putative binding sites identified in the 5′ UTR and the CDS of the gene, luciferase reporters holding the 5′ UTR, the entire CDS or a fragment of the CDS containing the most potent binding site were constructed. However, none of the reporters were affected by co-transfection with a miR-10a duplex (Figure S4B), suggesting that the identified sites were not functional miR-10a targets in this set-up. Further qRT-PCR analysis of *Lpo* transcripts in intestinal samples using primers in intronic and exonic sequence elements, revealed that the primary transcript of *Lpo* was upregulated in *miR-10a* KO samples to similar levels as the *Lpo* mRNA (Figure S4C and S4D), indicating that *Lpo* deregulation is transcriptional. Altogether, these results suggest that *Lpo* is not directly regulated by miR-10a via cognate interaction with target sites in the mRNA but instead that *Lpo* is regulated at the transcriptional level, probably by one or several primary targets of miR-10a.

### The miR-10a target Klf4 is upregulated in *miR-10a* KO mice and induces transcription of *LPO in vitro*

**Figure 4:**
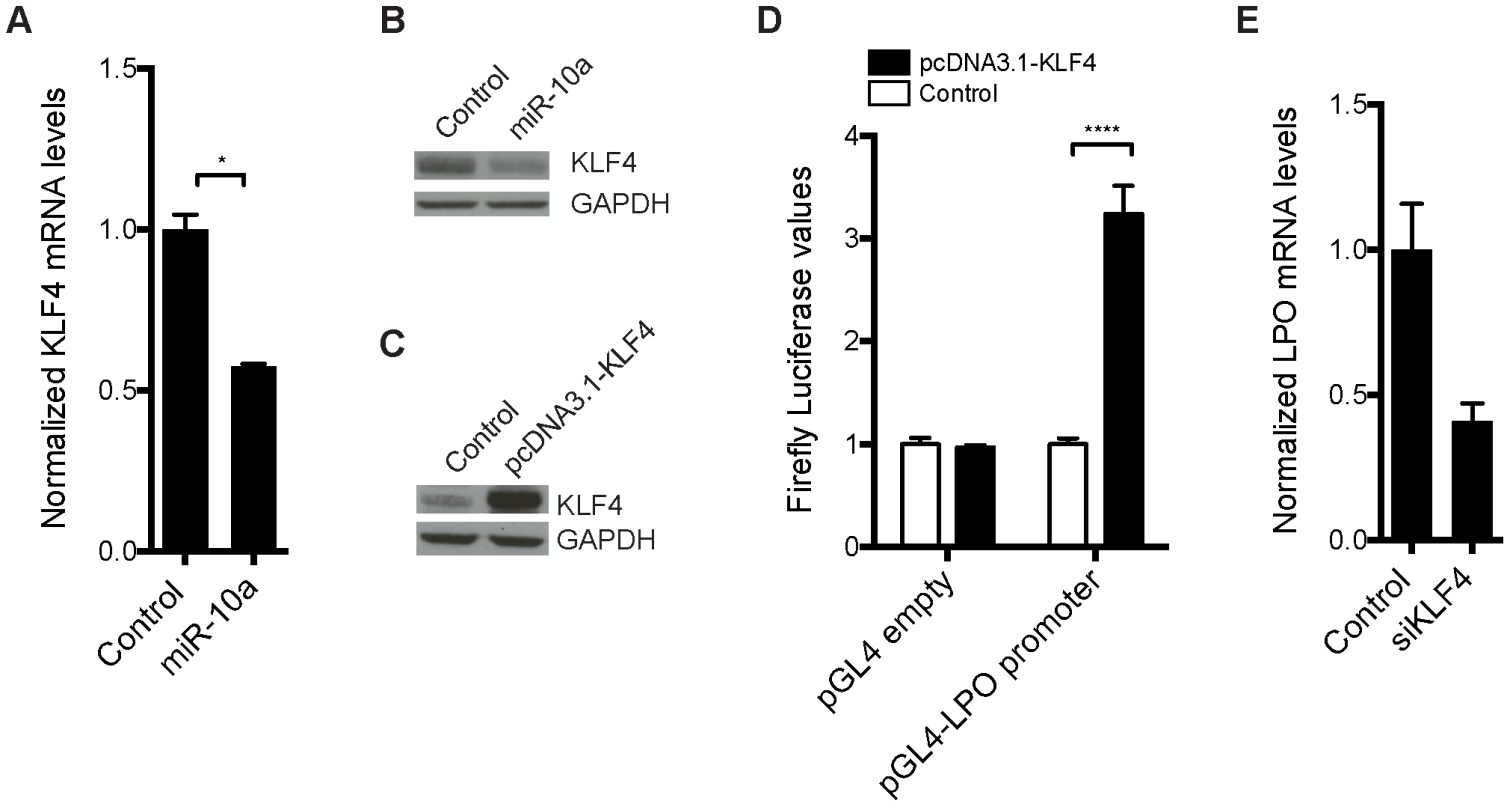
Transcription factor *KLF4* is regulated by miR-10a and can regulate the *LPO* promoter *in vitro*. HCT-116 cells were transfected with a miR-10a duplex or control for 72 h. (A) Relative mRNA levels of *KLF4* were measured by qRT-PCR and *ACTB* was used for normalization. Data are shown as mean ± S.D. of three replicates relative to the control and are representative of three independent experiments. * p<0.05 using a two-tailed *t*-test. (B) Protein levels in miR-10a or control transfected cells were assessed by Western-blot using antibodies against KLF4. GAPDH was used as loading control. (C) Western Blot showing the over expression from the pcDNA3.1-KLF4 vector. GAPDH was used as loading control. (D) Luciferase reporter assay in HCT-116 cells (24 h) with pGL4-luc2 holding part of the *LPO* promoter (1 kb upstream TSS) or the pGL4-luc2 empty vector co-transfected with a vector over-expressing KLF4 or a control vector (pcDNA3.1+). Data are shown as mean ± S.D. of three replicates relative to the pcDNA3.1 transfected control and are representative of eleven independent experiments. **** p<0.0001 using a two-tailed *t*-test. (E) HCT-116 cells were transfected with KLF4 siRNA for 48 h or 72 h. Relative mRNA levels of *LPO* were measured by qRT-PCR and *ACTB* was used for normalization. Data are shown as mean ± S.D. of three replicates relative to the control and are representative of five independent experiments.

We hypothesized that one or more transcription factors under miR-10a regulation could be responsible for enhancing *LPO* transcription. To identify such transcription factors, we scanned a 2 kb region upstream of the *LPO* transcription start site (TSS) using Consite and Transfac databases for transcription factor binding site motifs. From the obtained lists of transcription factors, we extracted those that were predicted as putative miR-10a targets by TargetScan [49] and pursued the analysis of one interesting candidate: Krüppel-like factor 4 (*Klf4*). Klf4, a zinc finger-type transcription factor primarily expressed in the gastrointestinal tract, is an important regulator of differentiation and cell growth arrest of the colonic epithelium and was previously shown to be regulated by miR-10a [50], [51]. Upregulation of KLF4 has formerly been observed in early stages of colon carcinoma compared to normal mucosal levels [52]. Using an *in vitro* setup, in the epithelial-like colon carcinoma cell line HCT-116, we demonstrated a 50% reduction of *KLF4* mRNA abundance upon transfection with miR-10a (Figure 4A). Importantly, miR-10a mediated repression of this target was also observed at the protein level (Figure 4B). These results confirm *KLF4* as a target of miR-10a as previously described [50].

To investigate the link between KLF4 and *LPO* we cloned a 1 kb fragment upstream of the TSS of *LPO*, holding core promoter elements, in front of a luciferase reporter. Co-transfection of this reporter vector with a KLF4 overexpression plasmid in HCT-116 cells resulted in a robust upregulation of luciferase activity, demonstrating the capacity of KLF4 to regulate *LPO* (Figure 4C and 4D). Using siRNA-mediated knockdown of KLF4 we further linked KLF4 to the regulation of LPO. Although these assays are complicated by a cell density-dependent expression of LPO, we found that knockdown of KLF4 in HCT-116 cells markedly reduced LPO mRNA levels (Figure 4E).

**Figure 5:**
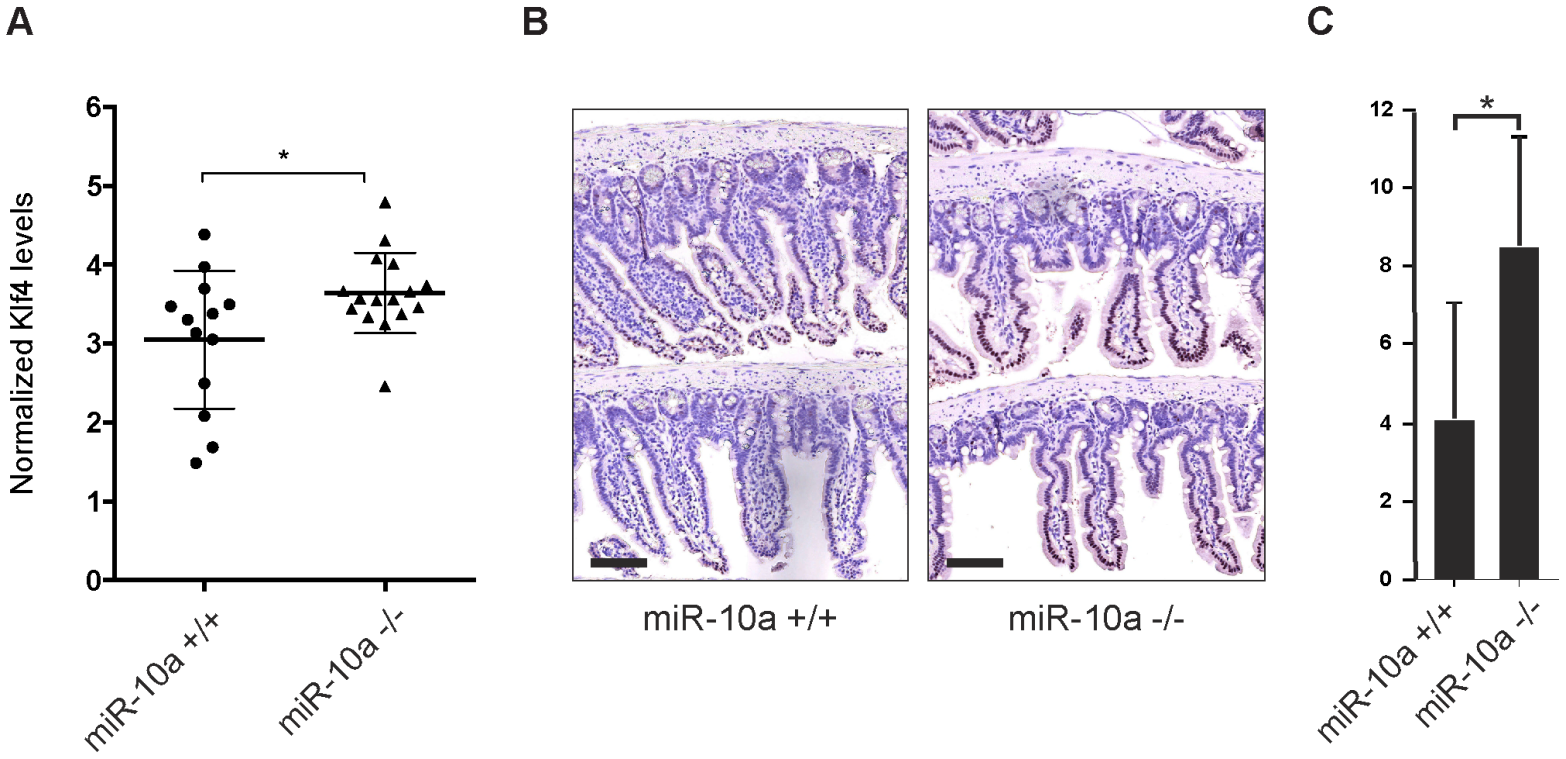
Klf4 is upregulated in *miR-10a* KO intestines. (A) mRNA levels of *Klf4* were measured by qRT-PCR, *Actb, Ubc, Hprt* and *36b4* were used for normalization. Data are shown as mean ± S.D. of *miR-10a* KO (n = 16) and WT (n = 13) samples relative to an average of the controls. * p<0.05 using a Mann-Whitney test. (B) Representative immunohistochemistry staining of Klf4 in *miR-10a* WT (n = 5) and KO (n = 8) intestine. Scale bar 100 µm (C) VisiomorphDP software scoring of Klf4 expression level estimated by distribution and staining intensity in Klf4 stained intestines of WT and miR-10a KO mice. * p = 0.019, students t-test.

Primary miRNA targets commonly show a relatively low degree of regulation on mRNA level after depletion of the miRNA. It can particularly be difficult to detect these changes in tissue samples that already present a considerable variation in mRNA expression between individuals and within different cell types of the tissue. In these types of experiments it is therefore crucial to use a sufficiently large population to reach statistical significance. In our microarray experiment we used intestinal RNA from only 6 animals (*miR-10a* KO; n = 3 and WT; n = 3) and we hypothesized that the limited population could account for the lack of detectable *Klf4* de-regulation. To increase the statistical power we measured intestinal *Klf4* mRNA levels from 16 miR-10a KO and 13 WT mice by qRT-PCR and used four housekeeping genes for normalization. The results showed a significant increase of *Klf4* mRNA levels in the miR-10a KO mice compared to the WT (Figure 5A). Consistently, Klf4 stainings demonstrated a marked increase of protein distribution and intensity in the mouse intestines of *miR-10a* KO compared to WT (Figure 5B and 5C). Specificity of Klf4 staining was verified by an independent antibody (Abcam; ab151733), which gave a similar expression pattern but resulted in an overall weaker staining (data not shown). Hence, our *in silico*, *in vitro* and *in vivo* results support the existence of a regulatory network linking miR-10a to *LPO* via KLF4 and potentially other transcription factors.

## Discussion

The enormous gene regulatory potential of miRNAs is well demonstrated by many studies showing that perturbed miRNA expression is capable of affecting diverse cellular functions and could ultimately cause disease, including cancer. However, it has been suggested that miRNAs are primarily important in fine-tuning mRNA expression and regulation executed by single miRNAs are in most cases not sufficient to account for pathological phenotypes [40], [53]. In line with this, KO of individual miRNAs in other animal models have revealed that most are devoid of obvious phenotypes in the absence of additional lesions or stresses [54], [55]. Particular interest in the miR-10 family members arises from their conserved genomic location in *Hox* clusters and the increasing amount of evidence for their implication in vertebrate biology and human disease [4]. Here we addressed the question of the direct causal effect of miR-10a in mammalian homeostasis, with a special focus on tumor development, by generating *miR-10a* null mice.

Despite the body of evidence suggesting a role for *miR-10* in *Hox* regulation, the *miR-10a^−/−^* mice showed an absence of major developmental defects in the posterior trunk. Nevertheless, these results are in agreement with the virtual lack of phenotypic differences upon inhibition or overexpression of miR-10 during zebra fish development [7]. In the case of *miR-10a^−/−^* mice, the lack of appreciable phenotypes could be explained by redundancy and functional compensation by miR-10b, which levels remained unaffected in *miR-10a* KO mice. A double inactivation of *miR-10a* and *miR-10b* would allow disambiguation of the *miR-10* role in mammalian development.

Interestingly, one gene, Lactoperoxidase, consistently showed an exceptionally high degree of deregulation in the intestines of *miR-10a* deficient mice and to our knowledge this is the first report correlating this gene in to a specific miRNA deficiency. Lpo is mainly described as an antibacterial agent [41], [56]–[58] exclusively found in mucosal surfaces, including colon epithelium [59] and exocrine secretions, like milk, tears, and saliva [60]. Importantly, apart from its antimicrobial and hydrogen peroxide detoxification activities, the role of LPO in carcinogenesis, particularly of the mammary gland, has been intensively studied [43]–[45], [61], [62]. In the reduction of peroxides, peroxidases can co-catalyze the oxidation of aromatic and heterocyclic amines into electrophilic metabolites with DNA binding capacity. In this way, Lactoperoxidase can catalyze the mutagenic activation of diverse endogenous and xenobiotic carcinogens, including natural [44], [62] and synthetic estrogens [63] as well as other synthetic or environmental arylamines [43], [45]. Lpo-mediated activation of such compounds to the derivative quinones and semiquinones, has been shown to induce the formation of the depurinating adducts N3Ade and N7Gua *in vitro* and *in vivo* [44], [45], [61]–[63]. Subsequent error-prone base excision repair mechanisms may lead to mutations that can be initiating events in breast, prostate and other types of cancer [47]. Furthermore, estradiol and its catechol metabolites have been shown to induce deletions and loss of heterozygosity in epithelial breast cells resulting in an oncogenic transformed phenotype [64].

Here we showed that Lpo is constitutively upregulated in the intestinal epithelium of *miR-10a^−/−^* mice and that when these mice were crossed with *Apc^Min^* mice, females displayed a significantly increased tumor burden in their intestinal epithelium. We therefore propose that, in our set-up, upregulated Lpo induces a mutagenic environment by increasing the oxidation of endogenous estrogens into their mutagenic derivatives, which subsequently leads to tumor formation. Consistently, this effect would be sex-dependent, since estrogens are intrinsically at higher concentrations in female relative to male mice. Similar to our observations, other genetic studies with *Apc^Min^* mice have shown that mutation of genes important for genome stability maintenance, generally lead to an increase in adenoma multiplicities [65]–[68].

Regarding the regulatory mechanism, the Lactoperoxidase mRNA does not contain a miR-10a target site in its 3′UTR. Functional interactions between microRNAs and target sites in other locations than the 3′UTR have been described before, including for miR-10a [69]–[74]. We therefore tested the functionality of putative miR-10a binding sites in the 5′UTR and coding region of *LPO*, however, our results led us to exclude the possibility of a direct posttranscriptional regulation of *LPO* by miR-10a. Instead we obtained evidence supporting a model where *LPO* expression is regulated by the primary miR-10a target KLF4, which is indeed over-expressed in the intestines of *miR-10a* KO mice. Putative binding sites for this transcription factor are present in the promoter of *LPO*, and transcriptional activation of *LPO* could be reproduced *in vitro* by over-expressing KLF4. Moreover, siRNA knockdown of KLF4 in HCT-116 cells resulted in downregulation of *LPO* expression. Of notice, although not tested experimentally, our bioinformatics approach identified other transcription factors representing primary miR-10a targets with putative binding sites in the *LPO* promoter, suggesting that additional factors may participate in the indirect regulation of *LPO* by miR-10a. This could, in part, explain the lack of detected variation in the expression levels of direct miR-10a targets (including *Klf4*) in our microarray experiments, since the occurrence of small degrees of deregulation of primary targets could have a measurable effect only upon convergence in a secondary node. Alternative explanations could be evoked due to the large variation inherent to tissue samples and the fact that the colon tissue comprises a variety of cell types, such as epithelial, luminal and muscular cells, which all have specific genetic programs, thus very different transcriptomes. A change in mRNA expression in one cell type could therefore be masked by the lack of change in another, which could be due to alternative miRNA-independent regulations or a potential rescue by miR-10b. Nevertheless, transcriptomic, bioinformatics and biochemical evidence allowed us to reveal a regulatory network where miR-10a can indirectly alter the levels of LPO through KLF4. Interestingly, intestinal miR-10a expression has previously been shown to be downregulated by microbiota in mice [75], and considering the antibacterial functions of Lpo in innate immunity, it is alluring to suggest that miR-10 could function as the sensor of immune stimuli in this environment where its downregulation would induce Lpo as an antibacterial mechanism in normal epithelium. However, having such a defense program constantly activated in the presence of estrogen, as would be the case in the *miR-10a^−/−^* female mice, would ultimately be damaging for the cells.

Our results are in contrast with reports of miR-10a upregulation in colon cancer samples [24], [25]. Such upregulation could correspond to a consequence rather than a cause of oncogenic transformation. This is enforced by the observation by Monzo et. al. showing an upregulation of miR-10a in stage II but not in stage I colon cancer samples [24], though the pathogenic role of miR-10a upregulation in advanced colon cancer remains to be elucidated. Moreover, Klf4 has been described to inhibit cell growth and play a tumor suppressive rather than an oncogenic role in colorectal cancer [51]. However, as suggested in our study, oncogenic downregulation of miR-10a might be a very early event promoting cellular transformation by a similar mechanism to the one previously described for Lpo in mammary carcinoma [44]. Furthermore, and as mentioned above, not only Klf4 but likely other primary targets of miR-10a are also involved in the regulation of Lpo.

In summary, here we present evidence that miR-10a, through a complex regulatory network involving the transcription factor Klf4, can contribute to tumor formation in female mice. By the indirect upregulation of Lpo levels in the intestinal epithelium, miR-10a deficiency in these mice creates an environment where estrogen could be transformed into potent depurinating mutagens that can ultimately lead to the initiation of cancer and tumor formation. Therefore we suggest that miR-10a may serve as a potential diagnostic marker for identifying groups of women that are at high risk of developing colorectal cancer.

## Materials and Methods

### Generation of *miR-10a neo/+* ES cells

The *miR-10a* targeting plasmid was constructed using standard recombineering techniques. R1-129 mouse ES cells were electroporated with the linearized targeting plasmid. G418 clones were selected and grown independently. Each clone was genotyped by southern blotting using the probes 5′/PacI and 3′/NsiI (Table S1) and *PacI*- or *NsiI*-digested DNA to verify 5′and 3′recombination respectively.

### Ethics statement

All mice were handled according to good animal handling practices and the animal experiments approved by the Danish Animal Experiments Inspectorate.

### Mice

*miR-10a* KO mice were generated as follows. ES cells from one of the three screened and correctly targeted clones were microinjected in C57BL/6 blastocyst and implanted in foster mothers. The resulting germline chimeras were bred with C57BL/6 (B6) to generate heterozygous mice for the targeted allele. F1 *miR-10a^neo/+^* males were crossed to a ubiquitously expressing *Cre* mouse line to eliminate the *neo* resistance cassette by Cre-LoxP recombination. Mice carrying the *miR-10a* floxed allele (*miR-10a* ^−^) were intercrossed with B6 mice for at least 7 generations before generating experimental cohorts of homozygous mutant and control mice. All mice were genotyped by PCR using tail-tip DNA and primers described in Figure 1A and Table S1, PCR conditions were 95°C for 5 min followed by 35 cycles of denaturation at 95°C for 30 sec, annealing at 56°C for 30 sec, extension at 72°C for 1 min and a final extension step at 72°C for 7 min.

*Apc^Min^* mice were obtained from The Jackson Laboratories and are described elsewhere [27], [28]. Only males were used for breeding with *miR-10a* ^+/+^ or *miR-10a* ^−/−^ mice. Genotyping of the *Min* allele was performed by PCR using the primers APC.fw, APC.rw and APC.Min under the same conditions described above. All primer sequences are shown in Table S1.

Mice had free access to food and water.

### Cell culture

HCT-116 cells were maintained in McCoy's 5A medium (Gibco) with 10% fetal bovine serum (Thermo Scientific) and 1% penicillin/streptomycin (Invitrogen), incubated at 37°C in 5% CO2.

### Quantitative RT-PCR

Total RNA was isolated from mice tissues with TRIzol (Invitrogen), as specified by the manufacturer, followed by DNase I treatment with the DNA-free kit (Applied Biosystems). HCT-116 cells were seeded in 6-well plates and reversely transfected with 50 nM of Allstars negative control (Qiagen; cat:1027281), a miR-10a duplex (Ambion; AM17100, PM10787) or siRNAs against *KLF4* (pool of two siRNAs with sequences: CCUUACACAUGAAGAGGCA[dT][dT], GUGGAUAUCAGGGUAUAAA[dT][dT], 25 nM each) using Lipofectamine2000 (Invitrogen). Cells were harvested 48 h or 72 h post-transfection for total RNA extraction using the miRNeasy Kit (Qiagen; 217004).

Mature miRNA specific qRT-PCR were performed using the TaqMan miRNA Assays (Applied Biosystems) for mmu/hsa-miR-10a and mmu/hsa-miR-10b; U6 small nuclear B non-coding RNA (RNU6B) and miR-184 were used as endogenous controls for normalization. For mRNA quantifications, first strand cDNA was synthesized from total RNA with Multiscribe Reverse Transcriptase (Applied Biosystems) and random hexamers. qRT-PCR was performed with the Fast SYBR Green master mix (Applied Biosystems) for *KLF4* and *Lpo* mRNA and gene specific primers are described in Table S3, *ACTB, Ubc, 36b4* and *Hprt* was used for normalization in mRNA relative quantifications. qRT-PCR for human *LPO* was performed with the TaqMan Gene Expression Assays (Applied Biosystems; AssayID: Hs00413417_m1) and *ACTB* (AssayID: Hs03023943_g1) was used for normalization. All PCR reactions were done in an Applied Biosystems 7900HT Fast Real-Time PCR System using SDS software. The comparative C_T_ method was used for relative quantification of all RNA species evaluated.

### Adenoma scoring, histopathological analysis and immunostaining

Mice were killed by CO_2_ asphyxiation or cervical dislocation. Then entire intestinal tract was removed and gently washed with cold PBS using a syringe before infusing them with 10% formalin; samples were kept at 4°C before being analyzed. After washing in PBS, intestines were opened lengthwise and examined under a dissection microscope equipped with a graduated ocular graticule at 20× magnification for polyp counting and measurement. The entire intestine was rolled and embedded in paraffin for further histopathological and histochemical evaluation. Paraffin embedded tissues were sectioned, rehydrated and stained using a standard H&E staining protocol. H&E stained sections were blindly examined for scoring of adenoma types and degree of dysplasia based on pathological criteria.

For immunohistochemical detection of LPO a polyclonal rabbit-anti-human LPO antibody (Thermo Fisher Scientific, Rockford, USA, PA1-46353) was used diluted 1∶200 where chromogen staining was achieved using the EnVision+ system (Dako, Glostrup, Denmark, K4003) in combination with NovaRED HRP substrate (VWR international, Herlev, Denmark, SK-4800). Stained sections were scanned using a NanoZoomer-2.0HT (Hamamatsu, Denmark) using 40× magnification. Scanned sections were evaluated, blinded in respect to genotype of mice, for low, medium or high Lpo expression. Comparison of Lpo expression between miR-10a^+/+^ (n = 5) and miR-10a^−/−^ (n = 10) mice was done by Pearson Chi-square test with exact probability and the analysis revealed a significant scoring difference (P≤0.006) between the two genotypes.

For immunohistochemical detection of Klf4 a polyclonal goat-anti-mouse Klf4 antibody (R&D; AF3158) was used diluted 1∶80 (2.5 µg/ml) where chromogen staining was achieved using the EnVision+ system (Dako, Glostrup, Denmark, K4003) in combination with NovaRED HRP substrate (VWR international, Herlev, Denmark, SK-4800), only 1/3 of normal hematoxylin stain was used to prevent a too strong blue nuclear signal that could bias the analysis. Stained sections were scanned using a NanoZoomer-2.0HT (Hamamatsu, Denmark) using 40× magnification. To quantify the expression level of Klf4, whole scanned sections were analyzed by the staining analysis software VisiomorphDP, which is part of the Visiopharm software package (Visiopharm, Hørsholm, Denmark). The formula A_positive_/(A_positive_+A_negative_)*(255-I_mean_) was used as a measure of the Klf4 expression. A_positive_ = area of positive cells nuclei, A_negative_ = area of Klf4 negative cell nuclei and I_mean_ = the mean intensity value (0–255, where the darkest colors have the lowest value) of the separating color band (ref.). Comparison of Klf4 expression between miR-10a^+/+^ (n = 5) and miR-10a^−/−^ (n = 8) mice was done by Students t-test and the analysis revealed a significant scoring difference (p = 0.019) between the two genotypes.

### Microarray analysis

Total RNA was extracted from colon samples of 4 months old, WT and *miR-10a* ^−/−^ female mice. Organs were collected as described in the previous section but after PBS washing, samples were frozen in liquid nitrogen and kept at −80°C until RNA was extracted. Four biological replicates of each genotype were analyzed on Affymetrix microarrays (GeneChip Mouse Gene 1.0 ST Array) at the Microarray Centre, Rigshospitalet, Copenhagen University Hospital as previously described [76].

Affymetrix probe set intensity of miR-10a KO and WT samples were preprocessed using the aroma package in BioConductor, including steps of background correction, normalization, and summarization by RMA (Robust Multichip Average) method.

We then applied a non-specific filtering step to exclude those genes showing low overall expression levels, as these genes were unlikely to show down- or upregulation after miRNA transfection. To do this, we required the interquartile range of probe set expression levels to be greater than the first quartile value of the interquartile range of expression levels for all probe sets. The duplicated probe sets mapped to the same genes and the probe sets without entrez gene annotation were also removed by this step. The remaining probe sets were subsequently mapped to gene symbols using the Affymetrix mogene10sttranscriptcluster.db annotation Package. Differentially expressed genes were identified by a moderated t-test with P-value less than 0.05 (452 transcripts and annotation as listed in Table S2), using Limma [77] package in Bioconductor. We defined three datasets: upregulated set (296 transcripts) with P-values≤0.05 and log FC>0, upregulated set (156 transcripts) with P-values≤0.05 and log FC<0, and no change set containing 323 transcripts from the gene set with P-value near 1.

### Western blot analysis

HCT-116 cells were seeded in 6-well plates and reverse transfected with 50 nM Allstars negative control (Qiagen; Cat: 1027281) or a miR-10a duplex (Ambion; AM17100, PM10787) using Lipofectamine2000 (Invitrogen). Cells were harvested 72 h post-transfection for protein extraction.

Cells or tissues were lysed and homogenized in RIPA buffer (150 mM NaCl, 0.5% DOC, 0.1% SDS, 1% Igepal, 50 mM Tris-HCl pH 8, 2 mM EDTA) containing 1 mM Pefabloc (Roche Applied Science) and 1× Complete Mini protease inhibitor mixture (Roche Applied Science). 20 µg of protein per lane from was separated on a 4–12% NuPAGE Bis-Tris gel (Invitrogen) for cell culture experiments and a 3–8% Tris-Acetate gel (Invitrogen) for mouse tissues, followed by transfer to a nitrocellulose membrane. Membranes were blocked in 5% milk for 40 min at room temperature and incubated over night with primary antibody at 4°C. Antibodies used were purchased from Santa Cruz: LPO ((H-60) sc-134848), KLF4 ((H-180): sc-20691).

### Luciferase reporter assays and vectors

A 1 kb sequence upstream the transcription start site of the *LPO* gene was cloned into pGL4-luc2 (Promega) using the following primer sequences (restriction sites *NheI* and *XhoI* are shown in lowercase letters): Fwd: 5′-TCgctagcTTTGCCTGGATTCATCAC-3′, Rev: 5′- TGctcgagCCTGAGCACATTTGTCCC-3′. The KLF4 over expressing vector was purchased from Addgene (pcDNA3.1-HA-KLF4-FL; plasmid #34593). HCT-116 cells were seeded in 96-well plates (15000 cells/well) and transfected the next day with 50 ng of pGL4-luc2-LPO promoter or pGL4-luc2 (empty) and 50 ng of pcDNA3.1-HA-KLF4-FL or pcDNA3.1+ (empty) using Lipofectamine2000 (Invitrogen). Luciferase expression was measured 24 h after transfection using the Dual-Glo luciferase assay (Promega, E2940)). For investigating the potential miR-10a binding sites part of the 5′UTR of *Lpo* was cloned into psi-CHECK-2 (Promega) using primers (restriction sites *NheI* is shown in lowercase letters): Fwd-5′UTR: 5′-GACgctagcACATCAACTGCTCCCTGACATCCT-3′, Rev-5′UTR: 5′-CGTgctagcTAAAGGACACACACACTCAGGCTCA-3′, and either the whole coding sequence (CDS) or a 609 bp sequence harboring the best miR-10a CDS binding site was cloned into a Firefly luciferase fusion vector pPK-CMV-F4 (PromoKine) using primer sequences (restriction sites *XhoI* and *HindIII* are shown in lowercase letters): Fwd-CDS: 5′-GTGctcgagACCATGGTTAAAGTGCTTCTGCATCTCC-3′ and Rev-CDS: 5′-CACaagcttTCCTTCACTGAGGCCCAGGGTG-3′ and Fwd-10a site: 5′- GTGctcgagACCATGGTTATCTGTCAGATTATCTCAAGC-3′ and Rev-10a site: 5′- CACaagcttTCCCCTGAGTCTTTAATTTAGGGTCACC-3′. HCT-116 cells were seeded in 96-well plates (15000 cells/well) and transfected the next day with 30 nM of miR-10a mimic (Applied Biosystems; AM17100, PM10787) or control (Qiagen; Allstars neg control: 1027281) and 100 ng of psi-CHECK-2-Lpo-5′UTR alone, or 100 ng of pPK-CMV-Lpo-CDS or pPK-CMV-Lpo-10a-site together with 10 ng pRL-TK (Promega; Renilla vector used for normalization) using Lipofectamine2000 (Invitrogen). Luciferase expression was measured 24 h after transfection using the Dual-Glo luciferase assay (Promega, E2940)).

## Supporting Information

Figure S1 Basic phenotypic characterization of *miR-10a* deficient mice. (A) *miR-10a^−^* allele segregation in the offspring of *miR-10a* ^+/−^ parents. (B) Weight of *miR-10a* ^+/+^ (WT males n = 17, females n = 24) and *miR-10a* ^−/−^ (KO, males n = 21, females n = 27) mice at 8 weeks of age. Detection of mature miR-10a (C) and miR-10b (D) by qRT-PCR in intestines of *miR-10a* ^+/+^ (WT) and *miR-10a* ^−/−^ (KO) mice. (E) Relative expression levels of *HoxB4* mRNA in intestines from mice of the indicated genotypes. Circles represent values from female mice and squares represent data from male mice. At least three different mice of each genotype were analyzed in three independent experiments. Results from one representative experiment are shown.
(TIF)

Figure S2 miR-10 expression levels in a panel of different organs. miR-10a (A) and miR-10b (B) expression levels in different organs of B6 mice as detected by qRT-PCR.
(TIF)

Figure S3 Relative expression levels of known miR-10a targets and genes with oncogenic or tumor suppressor potential relevant in intestinal tumorigenesis. mRNA levels were measured by qRT-PCR using specific primers for miR-10a known targets (A) *Usf2*, (B) *HoxA3*, (C) *HoxD10*, (D) *Hdac4*; Wnt/β-catenin pathway genes (E) *Cntb1*, (F) *Map3k7*, (G) c-*Jun*, (H) *Dvl3*, (I) *Sox2*, (J) *Ccnd1*, (K) c-*Myc*; Tgf-β signaling pathway genes (L) *Tgfbr2*, (M) *Smad6*, (N) *Smad7*, (O) *Pai1*; and other important intestinal oncogenes (P) k-*Ras*, (Q) *Egfr*, (R) *Cox2*. RNA samples from intestines of at least 4 different female mice were used in the analysis. All expression values are relative to the housekeeping mRNA levels of *Hprt*.
(TIF)

Figure S4 *Lpo* is a secondary target of miR-10a. (A) Predicted miR-10a binding sites in *Lpo* 5′UTR and CDS. (B) Luciferase reporter assay in HCT-116 cells (24 h) with constructs holding either the *Lpo* coding sequence (CDS), part of *Lpo* CDS including the best miR-10a putative binding site or the *LPO* 5′UTR co-transfected with a Renilla expressing vector along with a miR-10a duplex or control. Data are shown as mean ± S.D. of three replicates relative to the control and are representative of three independent experiments. n.s. using a two-tailed *t*-test. (C) *Lpo* primary transcript levels, using primers in *Lpo* intron 9, and (D) *Lpo* mature mRNA levels, using primers in *Lpo* exon 7 and 8, was measured by qRT-PCR *Actb* was used for normalization and values ± SD are shown relative to the first WT sample.
(TIF)

Table S1 Probes and primers used for mouse genotyping.
(PDF)

Table S2 Deregulated genes in the intestines of *miR-10a^−/−^* mice. 452 genes up- or downregulated with an FDR smaller than 0.05 before correction are shown. Genes are ordered by Fold change.
(XLSX)

Table S3 Primers used in qRT-PCR.
(PDF)
