# Supplementary figures and images for: Loss of miR-10a Activates Lpo and Collaborates with Activated Wnt Signaling in Inducing Intestinal Neoplasia in Female Mice
Source: PLoS Genet. 2013 Oct 24;9(10):e1003913. doi: 10.1371/journal.pgen.1003913 (PMC3812087; doi:10.1371/journal.pgen.1003913)

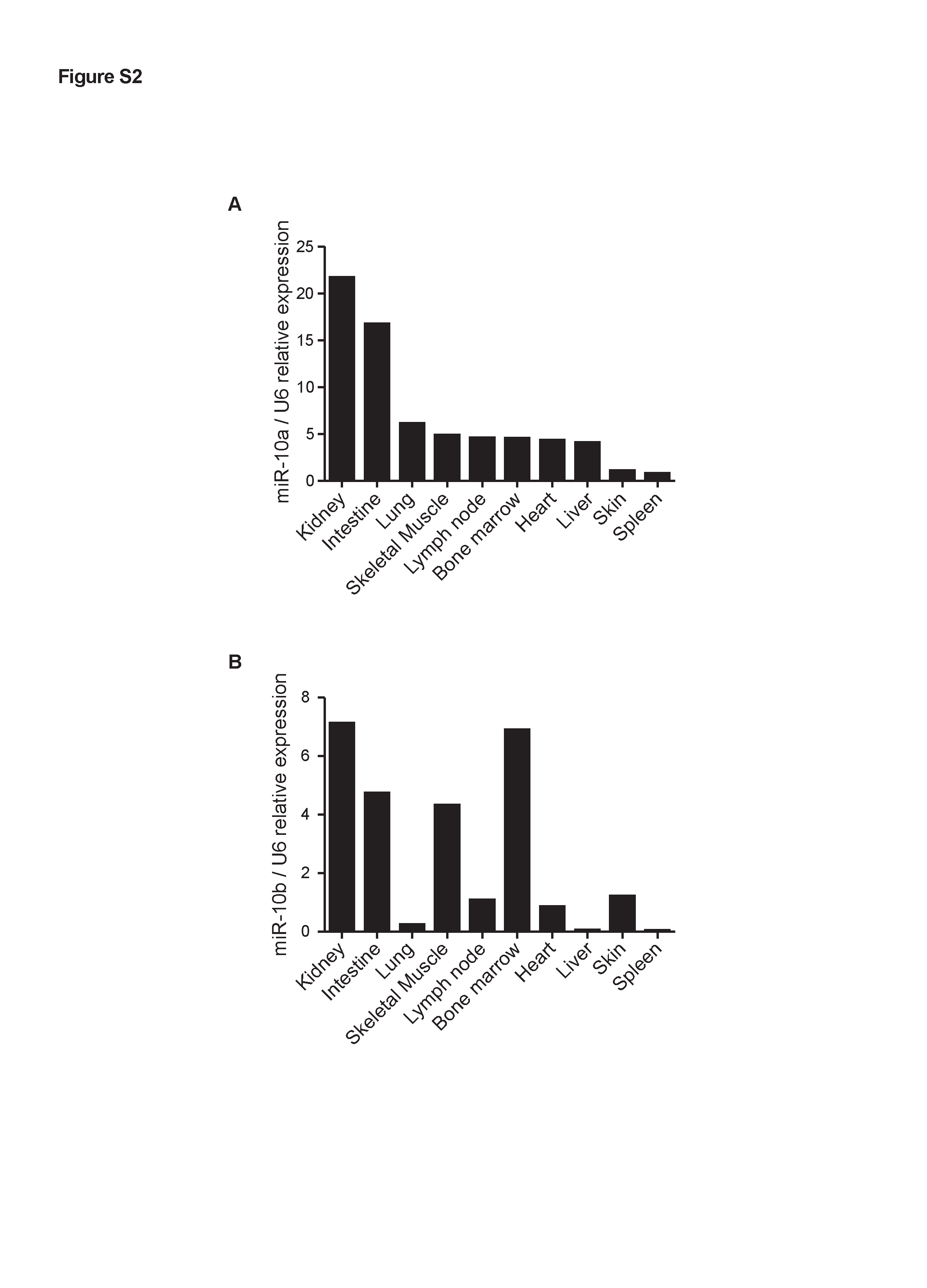

Supplement: Figure S2 — miR-10 expression levels in a panel of different organs. miR-10a (A) and miR-10b (B) expression levels in different organs of B6 mice as detected by qRT-PCR. (TIF) [file pgen.1003913.s002.tif]

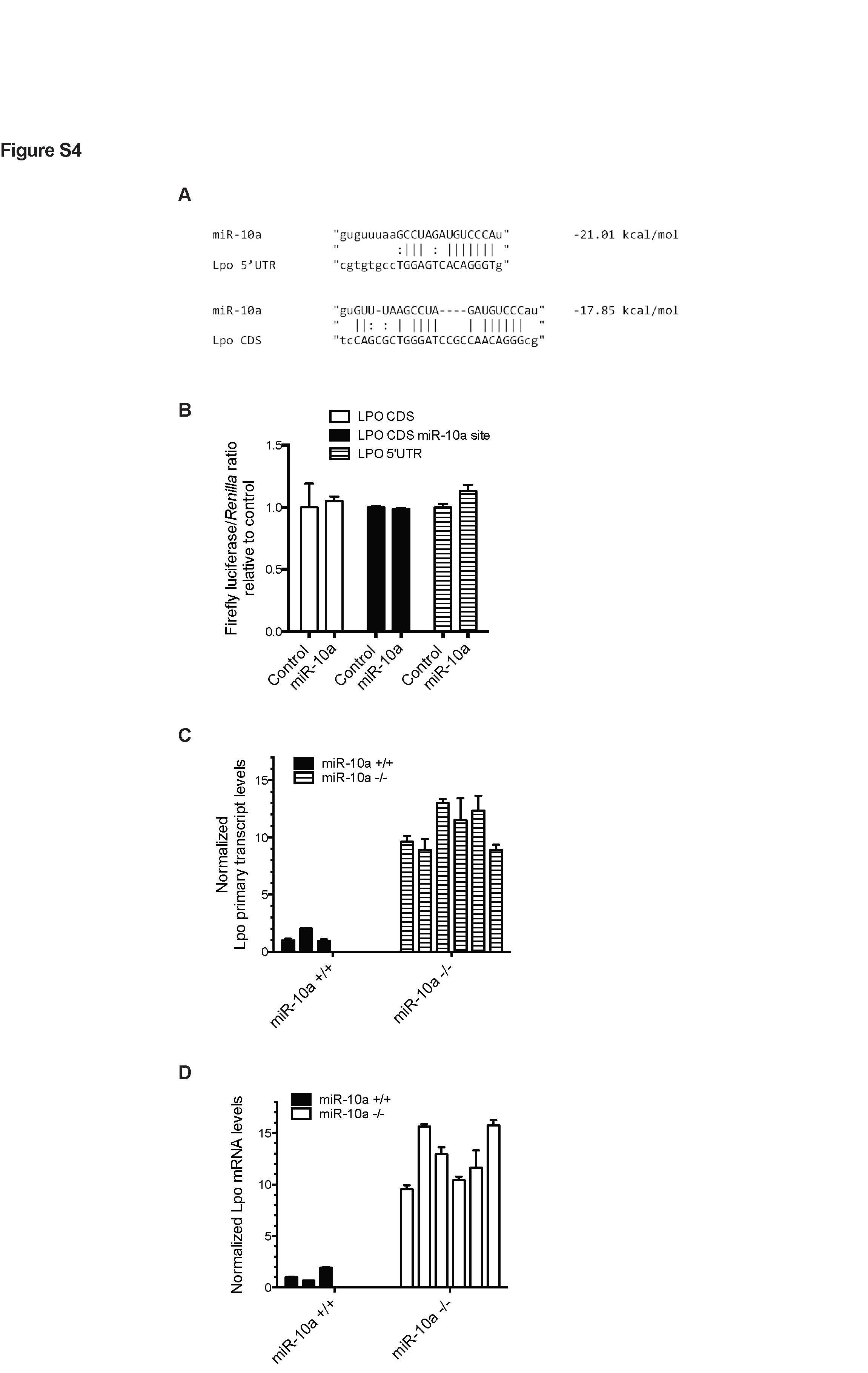

Supplement: Figure S4 — Lpo is a secondary target of miR-10a. (A) Predicted miR-10a binding sites in Lpo 5′UTR and CDS. (B) Luciferase reporter assay in HCT-116 cells (24 h) with constructs holding either the Lpo coding sequence (CDS), part of Lpo CDS including the best miR-10a putative binding site or the LPO 5′UTR co-transfected with a Renilla expressing vector along with a miR-10a duplex or control. Data are shown as mean ± S.D. of three replicates relative to the control and are representative of three independent experiments. n.s. using a two-tailed t-test. (C) Lpo primary transcript levels, using primers in Lpo intron 9, and (D) Lpo mature mRNA levels, using primers in Lpo exon 7 and 8, was measured by qRT-PCR Actb was used for normalization and values ± SD are shown relative to the first WT sample. (TIF) [file pgen.1003913.s004.tif]
