# Supplementary material for: Loss of miR-10a Activates Lpo and Collaborates with Activated Wnt Signaling in Inducing Intestinal Neoplasia in Female Mice
Source: PLoS Genet. 2013 Oct 24;9(10):e1003913. doi: 10.1371/journal.pgen.1003913 (PMC3812087; doi:10.1371/journal.pgen.1003913)
Supplement: Table S1 — Probes and primers used for mouse genotyping. (PDF) [file pgen.1003913.s005.pdf]

**Table S1. Probes and primers used for mouse genotyping**

| Name | Sequence |
|---|---|
| 5’probe/PacI | 5’- AAACCTACCC………………. GAACTAGGGC-3’ |
| 3’probe/NsiI | 5’- CCCGCAATGA………………. ATGGCCCAGG-3’ |
| L_chkinsrtmiR10a.5 | 5’- AAAGTTGAAACCCCTTGTAATCC-3’ |
| R_chkinsrtmiR10a.5 | 5’- ACATTTCCCCGAAAAGTGC-3’ |
| 10a.internal | 5’- TGAGCGGAGTGTTTATGTCAA -3’ |
| 10a.fwd | 5’- CATTCAGATCACACCCAAAAGA-3’ |
| 10a.rev | 5’- GGCAGTCACTGGATTGTCCT-3’ |
| APC.fwd | 5’-CACCGGAGTAAGCAGAGACA-3’ |
| APC.min | 5’-TTCTGAGAAAGACAGAAGTTA-3’ |
| APC.rev | 5’-GAACTCGGTGGTAGAAGCAG-3’ |
