# Supplementary material for: Loss of miR-10a Activates Lpo and Collaborates with Activated Wnt Signaling in Inducing Intestinal Neoplasia in Female Mice
Source: PLoS Genet. 2013 Oct 24;9(10):e1003913. doi: 10.1371/journal.pgen.1003913 (PMC3812087; doi:10.1371/journal.pgen.1003913)
Supplement: Table S3 — Primers used in qRT-PCR. (PDF) [file pgen.1003913.s007.pdf]

Table S3. qPCR primers

Gene	Forward primer sequence	Reverse primer sequence	Species
Hprt	5’-CAGGCCAGACTTTGTTGGAT-3’	5’-TTGCGCTCATCTTAGGCTT-3’	mmu
HoxB4	5’- AAAGAGCCCGTCGTCTACC-3’	5’- GCGTCAGGTAGCGATTGTAGT-3’	mmu
Usf2	5’- TTCAGACAGGCACACAGAGG-3’	5’- GATTTTGGAAAGCTGGACGA-3’	mmu
Hdac43	5’- TGGTATGGGAAGACACAGC-3’	5’- ATCTTTGGCGTCGTACATTC-3’	mmu
HoxA3	5’- AGGTAGCGGTTGAAGTGGAA-3’	5’- TTCCCTGGATGAAAGAGTCAA-3’	mmu
HoxD10	5’-AGGAGCTGCCTGGCTGAGGT-3’	5’-CGCTCGCGGGTGAGGTACA-3’	mmu
Dvl3	5’-GTGGTAACATGGCCAACC-3’	5’-ACTGGTAAGGGAAAGCCATAG-3’	mmu
Map3K7	5’-ACCAGCACAGGCTCATTC-3’	5’-TGACTCCAAGCGTTTAATAGTG-3’	mmu
Myc	5’-TGAAGGCTGGATTTCCTTTG-3’	5’-ACGGAGTCGTAGTCGAGGTC-3’	mmu
cJun	5’-AGTAGCCCCCAACCTCTTTG-3’	5’-ACAGGGGACACAGCTTTCAC-3’	mmu
Axin2	5’-CAGCAGAGGGACAGGAAC-3’	5’-TTGGCTCTTTGTGATCTTCTG-3’	mmu
Sox2	5’-GCACATGAACGGCTGGAGCAACG-3’	5’-TGCTGCGAGTAGGACATGCTGTAGG-3’	mmu
Ccnd1	5’-TTGACTGCCGAGAAGTTGTG-3’	5’-GAGCTTGTTCACCAGAAGCA-3’	mmu
Ctnnb1	5’-TTGCTCAACAAAACAAACGTG-3’	5’-ACCACTGGCCAGAATGATGA-3’	mmu
Tgfbr2	5’-GCCGTGTGGAGGAAGAAC-3’	5’-AGAGTGAAGCCGTGGTAGG-3’	mmu
Smad6	5’-TCTGCGGGCCAGAATCAC-3’	5’-AGGAGGAGACAGCCGAGAATAG-3’	mmu
Smad7	5’-CCCTCCTCCTTACTCCAGATAC-3’	5’-ACAGCCTGCAGTTGGTTTG-3’	mmu
Pai1	5’-TCAACTACACTGAGTTCACC-3’	5’-AGTTCCACAACGTCATACTC-3’	mmu
Egfr	5’-GAACAAAGCAACATGGTCAG-3’	5’-CCAGTTTATTGTGTTTGCGTAG-3’	mmu
Cox2	5’-CTCAGCCAGGCAGCAAATC-3’	5’-TGGTCAAATCCTGTGCTCATAC-3’	mmu
kRas	5’-CCTGGTAGGGAATAAGTGTG-3’	5’-TCAACACCCTGTCTTGTC-3’	mmu
Lpo intron 9	5'-GCCCTCTTCCCGCATTCTGCC-3'	5'-TCAAGTCCCTGCAAGGGGGC-3'	mmu
Lpo mRNA	5'-ACCACGACATGGACTTTGCCCC-3'	5'-AGGCATGCACTTCCCCTGAGTC-3'	mmu
ACTB/Actb	5'-CTCCCCGGGCTGTATTCC-3'	5'-CCTCTCTTGCTCTGGGCC-3'	hsa, mmu
KLF4	5'-GGGCTGCGGCAAAACCTACACA-3'	5'-CCATCCACAGCCGTCCCAGTCA-3'	hsa
